# Psychosocial Interventions for Improving Treatment Adherence in Tuberculosis Patients: A Scoping Review of Evidence-Based Approaches

**DOI:** 10.3390/arm94030032

**Published:** 2026-05-15

**Authors:** Rana Abdullah Bin Qamar, Henrique Pereira, Felipe Alckmin-Carvalho

**Affiliations:** 1 Department of Psychology, Faculty of Economics, Administrative and Social Sciences, Istanbul Rumeli University, 34570 Istanbul, Türkiye; 2 Department of Psychology and Education, Faculty of Social and Human Sciences, University of Beira Interior, 6200-209 Covilhã, Portugal; hpereira@ubi.pt (H.P.); felipe.carvalho@ubi.pt (F.A.-C.); 3 Research Center in Sports Sciences, Health Sciences and Human Development (CIDESD), 5001-801 Vila Real, Portugal

**Keywords:** tuberculosis, psychosocial interventions, treatment adherence, scoping review, mental health

## Abstract

**Highlights:**

**What are the main findings?**

- Mental health comorbidities, especially depression and anxiety and, in MDR-TB, psychosis, are common among people with TB and are closely linked to socioeconomic vulnerability and stigma.
- Evidence-based psychosocial interventions, including TB psychoeducation, Motivational Enhancement Therapy, CBT, ACT, and multicomponent support, can improve psychological outcomes and may strengthen treatment adherence in both drug-susceptible and drug-resistant TB.

**What are the implications of the main findings?**

- TB services should routinely integrate mental health screening and brief, scalable psychosocial care to address stigma, distress, and other adherence barriers alongside biomedical treatment.
- Adherence may improve further when psychological and educational strategies are combined with social protection, such as peer support and practical assistance, using task-shifting models where feasible in resource-constrained settings.

**Abstract:**

This scoping review synthesized evidence on the psychosocial burden of tuberculosis (TB) and on evidence-based psychosocial interventions aimed at improving treatment adherence. Specifically, it examined: (a) the most frequent mental health problems associated with TB; (b) the main barriers to adherence; (c) the components and effects of psychosocial interventions; and (d) gaps in the literature and directions for future research. Bibliographic searches were conducted in PubMed and Scopus, covering articles published between 2005 and 2025. Nineteen studies met the inclusion criteria. Depression and anxiety were the most frequently reported mental health problems, while psychosis appeared mainly in multidrug-resistant TB (MDR-TB) populations. Across studies, stigma, fear of transmission, socioeconomic disadvantage, treatment duration, and medication side effects emerged as major barriers to adherence. Evidence-based interventions—including psychoeducation, motivational enhancement therapy, cognitive behavioral therapy, acceptance and commitment therapy, and multicomponent psychosocial support—were associated with improved psychological outcomes and, in several studies, better adherence-related indicators. Overall, the evidence suggests that psychosocial distress is common among people with TB and may compromise treatment engagement. Integrating psychosocial and mental health support into TB services may therefore strengthen adherence and improve patient-centered outcomes, although more rigorous and context-sensitive research is still needed.

## 1. Introduction

Tuberculosis (TB) is an infectious disease caused by Mycobacterium tuberculosis, most often affecting the lungs and transmitted through airborne particles. It remains a major global public health challenge. According to the World Health Organization, an estimated 10.8 million people developed TB in 2023 and approximately 1.25 million died from the disease [1]. Although TB is preventable and curable, it continues to impose a substantial burden on individuals, families, and health systems, particularly in low- and middle-income settings.

Standard treatment for drug-susceptible TB usually lasts 6 months, whereas treatment for multidrug-resistant TB (MDR-TB)—caused by strains resistant to at least isoniazid and rifampicin—may extend to 18–24 months depending on the regimen [1]. Poor adherence remains a major challenge, with non-adherence estimates ranging from 20% to 50% across studies [2,3]. The consequences of poor adherence include delayed sputum culture conversion, treatment failure, relapse, prolonged infectiousness, development of drug resistance, and increased morbidity and mortality [4,5]. Patients who do not adhere to treatment may take substantially longer to achieve culture conversion and often require longer treatment courses [5].

Treatment adherence is shaped by interacting determinants across multiple domains, including socioeconomic conditions (e.g., poverty, limited social support, transport costs), health-system factors (e.g., poor patient–provider communication, distance to treatment centers), condition-related factors (e.g., side effects, symptom burden), therapy-related factors (e.g., regimen complexity, pill burden), and patient-related factors (e.g., limited knowledge, fear of stigma, substance use, and mental health problems) [2,3]. Among these, the psychological burden associated with TB has emerged as a particularly important determinant of treatment engagement and outcomes, yet it is still insufficiently addressed in routine TB care [6].

There is growing evidence that common mental disorders, especially depression and anxiety, are highly prevalent among people with TB, with estimates often ranging from approximately 40% to 60%, and with an even greater burden among MDR-TB populations [7,8,9]. The relationship between TB and mental health is bidirectional and complex. On one hand, the experience of being diagnosed with and treated for TB can precipitate or worsen mental health problems through several pathways: anticipated and enacted stigma may lead to social withdrawal and internalized shame; prolonged treatment regimens (6 months for drug-susceptible TB and up to 18–24 months for MDR-TB) often involve adverse effects such as fatigue, hepatotoxicity, and, in the case of cycloserine, neuropsychiatric symptoms ranging from depression to psychosis; loss of income from work absence and out-of-pocket costs may compound poverty and family conflict; and isolation from family and community can intensify loneliness and hopelessness [6,7]. On the other hand, pre-existing or comorbid mental disorders may compromise help-seeking and adherence by reducing motivation, impairing concentration and memory for daily medication routines, increasing risky behaviors such as alcohol or drug use, and weakening the patient–provider relationship; depression and anxiety in particular have been linked to delayed diagnosis, missed doses, treatment interruption, and poorer microbiological outcomes [10,11,12]. These mutually reinforcing pathways have been described as a TB–depression syndemic, in which biological, psychological, and social vulnerabilities cluster and amplify each other in low-resource and high-stigma settings [6]. TB diagnosis and treatment can generate psychological distress through stigma, social isolation, economic hardship, and medication side effects, while pre-existing or comorbid mental health problems can undermine help-seeking, adherence, and treatment outcomes [6,12]. Patients with depression or anxiety are more likely to report difficulties adhering to treatment and to experience poorer outcomes [10,11].

Considering the multidimensional factors that hinder TB treatment adherence and the substantial psychological burden experienced by people with TB, biomedical interventions alone may be insufficient to address all barriers to optimal treatment outcomes [13]. The End TB Strategy emphasizes patient-centered and integrated approaches that address the psychosocial determinants of TB. In the present review, psychosocial interventions are defined as non-pharmacological, structured strategies that address psychological, behavioral, and social determinants of health by combining one or more of the following components: psychoeducation, individual or group counseling, evidence-based psychotherapies (such as cognitive behavioral therapy, acceptance and commitment therapy, or motivational enhancement therapy), peer or family support, and material or social assistance [14,15,16]. Psychosocial interventions—including counseling, psychotherapy, peer support, psychoeducation, and material assistance—have shown promise in improving treatment engagement and outcomes [16]. Nevertheless, the evidence base remains heterogeneous, with substantial variation in intervention components, delivery models, target populations, and outcome measures across studies. A scoping review is particularly timely now because the End TB Strategy [13] and the WHO mhGAP framework [17] have explicitly called for the integration of mental health and psychosocial care into TB programs, while the number of primary trials and qualitative studies in this area has expanded substantially over the past five years [18,19,20,21]. Mapping this rapidly growing and methodologically heterogeneous literature is needed to clarify what works, for whom, and under which conditions, before further investment in larger-scale trials and program implementation.

The purpose of this scoping review was to synthesize the available evidence on psychosocial interventions that may enhance treatment adherence among people with TB. Specifically, this review aimed to identify: (a) the most frequent mental health problems associated with TB; (b) the main barriers to treatment adherence; (c) the components and effects of evidence-based psychosocial interventions; and (d) gaps in the literature and directions for future research. To guide the synthesis, the following research questions were formulated: (1) What are the most frequent mental health problems experienced by adolescents and adults undergoing active TB treatment, including drug-susceptible and multidrug-resistant TB? (2) What are the main psychosocial barriers to TB treatment adherence reported in the literature? (3) Which psychosocial interventions—pertaining to their components, theoretical frameworks, and delivery models—have been evaluated for improving TB treatment adherence and related psychological outcomes, and what is their reported effectiveness? (4) What gaps and methodological limitations remain in the existing evidence base, and what directions should future research and clinical practice take to strengthen psychosocial care in TB programs?

## 2. Materials and Methods

### 2.1. Study Design and Approach

This study was conducted as a scoping review, an approach that is especially suitable for organizing and interpreting broad, heterogeneous bodies of literature. Scoping reviews are particularly useful when studies vary in methods, populations, and outcome measures, and when the objective is to map key concepts, summarize existing evidence, identify knowledge gaps, and clarify priorities for future research. Accordingly, this review aimed to provide a comprehensive overview of psychosocial burden and psychosocial interventions in active TB treatment, highlighting what is known, what remains uncertain, and what may inform future research and TB care practice. This review is reported in accordance with the PRISMA-ScR guideline [22].

During the preparation of this manuscript, the authors used ChatGPT (OpenAI, GPT-5.3) to assist with translating portions of the text into English and formatting the manuscript according to the submission guidelines of Advances in Respiratory Medicine. The tool was used only for language and editorial assistance. All generated text was carefully reviewed, edited and verified by the authors, who take full responsibility for the accuracy and integrity of the final manuscript. In addition, prior to resubmission, the entire manuscript underwent a comprehensive round of human-led language revision and copyediting by the authors, with attention to academic register, terminological precision, internal consistency, and accuracy of citations and reference formatting. All sentences originally produced with the assistance of ChatGPT were reviewed, refined, and, where appropriate, rewritten by the authors. No content, interpretation, or scientific conclusion in this manuscript was generated solely by an artificial intelligence tool.

### 2.2. Search Strategy

An extensive bibliographic search was undertaken in PubMed and Scopus to identify studies addressing psychosocial burden and psychosocial interventions in active tuberculosis (TB) treatment. PubMed and Scopus were selected for two reasons. First, both databases provide broad and complementary coverage of biomedical and health-sciences literature relevant to TB and to mental health: PubMed offers comprehensive coverage of MEDLINE-indexed clinical and public-health journals, while Scopus expands coverage to include nursing, social sciences, and a substantial proportion of titles indexed in Web of Science [23,24]. Second, PsycINFO, Web of Science, and Google Scholar were considered but ultimately not selected: at the time of the search, document-level access to PsycINFO and Web of Science was not available through the institutional credentials used for this review, and Google Scholar was not used as a primary database because it lacks structured Boolean indexing, returns inconsistent and non-reproducible result sets, and is therefore not recommended as a sole or primary source for systematic and scoping reviews [24]. To minimize the risk of missing relevant studies, the reference lists of all included reviews and primary studies were hand-searched for additional eligible records. The search covered articles published between January 2005 and December 2025. Studies were screened based on predefined eligibility criteria, focusing on empirical research examining psychosocial determinants, mental health outcomes, and structured psychosocial interventions during active TB treatment. Full database-specific Boolean search strategies are provided in Table 1.

### 2.3. Selection of Studies

**Figure 1:**
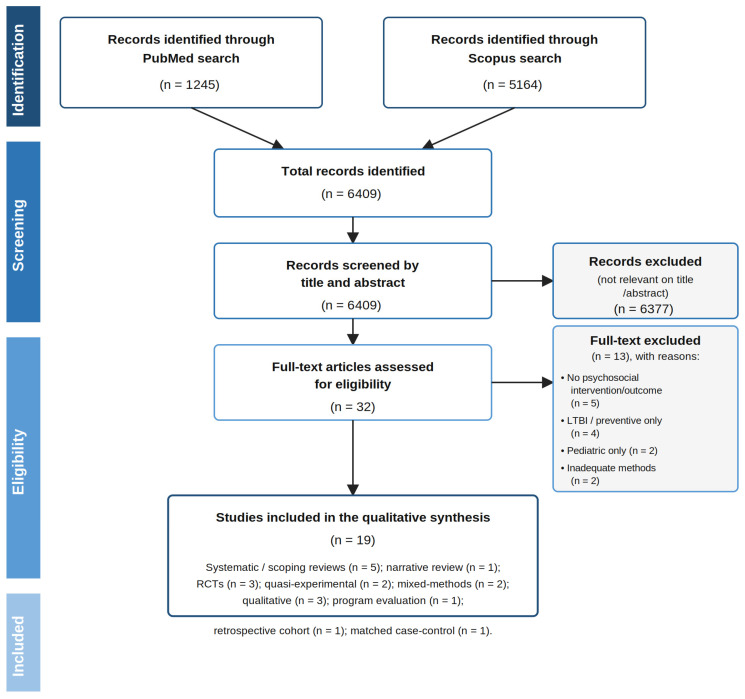
PRISMA-ScR flow chart of the study selection process. Adapted from Tricco et al. [22]. Search period: January 2005–December 2025.

All records retrieved from the electronic database searches were exported to a reference-management file, and duplicates were removed before screening. The first author conducted the initial screening of titles and abstracts and performed the full-text review of potentially relevant studies. The third author independently double-checked the articles selected by the first author against the predefined inclusion and exclusion criteria. Any disagreements during screening or full-text assessment were resolved through discussion between the two authors. The overall study selection process is presented in the PRISMA-ScR flow diagram (Figure 1), which documents the identification, screening, eligibility assessment, and inclusion stages, together with reasons for full-text exclusion.

### 2.4. Inclusion and Exclusion Criteria

#### 2.4.1. Inclusion Criteria

Articles of different designs (quantitative, qualitative, and mixed-method primary studies, as well as systematic and scoping reviews and meta-analyses) addressing:

- Adolescents’ and adults’ (≥15 years) adherence to active TB treatment (drug-susceptible TB and multidrug-/rifampicin-resistant TB [MDR/RR-TB]) and related psychopathology.
- Psychosocial variables associated with adherence to treatment for active tuberculosis.
- The efficacy or effectiveness of psychosocial interventions designed to improve adherence to active tuberculosis treatment in any healthcare or community TB-care setting.

#### 2.4.2. Exclusion Criteria

- Studies focused exclusively on latent TB infection (LTBI) or TB preventive therapy (TPT) without active TB.
- Studies that did not include psychosocial interventions or psychosocial outcomes of interest.
- Studies including pediatric populations only.
- Studies with inadequate methodological description.

### 2.5. Quality Evaluation and Integration

Quality appraisal was conducted using the tool most appropriate for each study design (AMSTAR 2 for systematic reviews, the Cochrane Risk of Bias tool for randomized trials, the MMAT for mixed-method and qualitative studies, and the JBI Scoping Review Checklist for Scoping Reviews). The first author conducted the initial quality assessment of all included studies, and the third author independently reviewed the appraisals using the selected tools. Any discrepancies were resolved through discussion between the authors until consensus was reached.

## 3. Results

The study selection process is summarized in Figure 1. Database searches conducted in PubMed (*n* = 1245) and Scopus (*n* = 5164) yielded a total of 6409 records. After title and abstract screening, 32 articles were retained for full-text assessment. Of these, 19 studies met the predefined inclusion criteria and were included in this review, while 13 full-text articles were excluded because they did not include psychosocial interventions or outcomes of interest (*n* = 5), focused exclusively on latent TB infection or preventive therapy (*n* = 4), included pediatric-only populations (*n* = 2), or provided inadequate methodological description (*n* = 2). A high-resolution version of this figure is provided in Supplementary Figure S1.

### 3.1. Mental Health Burden Among Patients with Tuberculosis

Meta-analytic evidence consistently indicates a high burden of mental health comorbidity in TB populations. Table 2 summarizes pooled prevalence estimates from systematic reviews and meta-analyses. Depression was the most consistently reported outcome: Duko et al. [9] estimated a pooled prevalence of 45.19% (95% CI [38.04, 52.55]) across 25 studies involving 4903 participants with TB. Among MDR-TB populations, Alene et al. [7] reported pooled prevalences of 25% for depression, 24% for anxiety, and 10% for psychosis. Alene et al. [7] also noted that psychosis was often described as a potentially reversible adverse effect during MDR-TB treatment, particularly in the context of second-line drugs such as cycloserine. In subgroup analyses, Duko et al. [9] reported a higher pooled prevalence of depression in women (51.54%) than in men (45.25%), and a somewhat higher prevalence in MDR-TB than in drug-susceptible TB (52.34% vs. 43.47%), although the latter difference was not statistically significant. Table 2 presents these pooled estimates together with their 95% confidence intervals and heterogeneity statistics (I^2^).

### 3.2. Overview of Included Studies

This review included 19 studies meeting the eligibility criteria. To avoid double-counting across overlapping designs (e.g., mixed-method cohort studies), each paper was classified into a single, mutually exclusive methodological category. The evidence comprised systematic/scoping reviews (*n* = 5) [7,9,25,26,27] and one narrative/perspective review (*n* = 1) [6]. Primary research included randomized/controlled trials (*n* = 3) [28,29,30], quasi-experimental studies (*n* = 2) [31,32], mixed-method studies (*n* = 2) [18,33], qualitative studies (*n* = 3) [21,34,35], one program evaluation (*n* = 1) [36], one retrospective cohort study (*n* = 1) [19], and one matched case–control study (*n* = 1) [37].

The included studies spanned multiple regions and more than ten countries. The highest representation came from India [18,28], China [30,33], and Indonesia [31,32]. Additional single-country primary studies were conducted in Ethiopia [29], Brazil [37], Peru [34], Kazakhstan [36], Zambia [35], Romania [19], and Portugal [21]. The remaining six studies were multinational evidence syntheses or conceptual reviews drawing on data from multiple countries and regions [6,7,9,25,26,27].

Publication years ranged from 2005 to 2025, with 15 of the 19 studies (approximately 79%) published between 2015 and 2025, indicating increasing attention to psychosocial dimensions of tuberculosis care. 

Table 3 presents an overview of the included studies, outlining the author, paper, country, study type, sample characteristics, research objectives, instruments and measures, interventions, and main results.

### 3.3. Effectiveness of Psychosocial Interventions

Table 4 presents a synthesis of primary intervention studies evaluating psychosocial strategies aimed at improving treatment adherence and mental health outcomes among patients with tuberculosis. The included studies examined a range of interventions, including Cognitive Behavioral Therapy (CBT), Acceptance and Commitment Therapy (ACT), Psychoeducation, Motivational Enhancement Therapy (MET), and Health Belief Model-based counseling. Across studies, psychosocial interventions were generally associated with improvements in depression, anxiety, psychological distress, and treatment adherence compared with usual care or control conditions. Cluster randomized trials [29,30] demonstrated statistically significant reductions in non-adherence and psychological symptom scores, while quasi-experimental and controlled studies reported improvements in depression severity and treatment success rates. Across studies, structured psychosocial interventions were associated with improvements in both mental health outcomes and adherence-related indicators in TB care settings.

Studies focusing on multidrug-resistant tuberculosis (MDR-TB) populations also highlighted the potential role of psychosocial support in promoting treatment continuity. Long-term participation in facilitated patient support groups was associated with sustained engagement in care and low treatment-interruption rates, alongside perceived adherence benefits among participants [34]. Similarly, the implementation of a structured psychosocial support (PSS) program in Kazakhstan was associated with reductions in default rates and improvements in dose adherence during the program rollout period [36]. Although these findings derive from non-randomized and programmatic evaluations, they suggest that comprehensive psychosocial support may contribute positively to adherence outcomes among MDR-TB patients.

### 3.4. Implementation Context and Delivery Models

Table 5 summarizes the structural and delivery characteristics of primary psychosocial intervention studies included in this review. The table outlines intervention settings, provider types, session frequency, and overall duration. Across studies, delivery models varied according to local healthcare infrastructure and available workforce capacity. Interventions were implemented in both community-based and clinic-based settings and were delivered by a range of providers, including multidisciplinary teams, specialist psychologists, general practitioners, and nurses. Session frequency and duration often reflected the clinical phases of TB treatment, with more intensive contact occurring during the early months of therapy.

### 3.5. Overall Quality of Included Studies

The methodological quality of the included studies varied across designs. Randomized and cluster-randomized trials generally demonstrated low risk of bias and were rated as high quality. Quasi-experimental and program-evaluation studies were of moderate quality, primarily because of non-randomized designs and limited control of confounding. Systematic and scoping reviews ranged from moderate to high quality according to AMSTAR 2 and JBI Scoping Review Checklist criteria. Qualitative and mixed-method studies met core MMAT standards but frequently lacked reflexivity reporting and formal triangulation. Observational cohort and case–control studies were of moderate quality, with appropriate statistical methods but limited capacity for causal inference. Conclusions and recommendations in this review were therefore weighted toward higher-quality interventional and meta-analytic evidence, while findings from observational and qualitative studies were used to contextualize implementation, stigma, and patient-experience dimensions.

## 4. Discussion

This scoping review synthesized evidence on psychosocial interventions designed to enhance treatment adherence among people with tuberculosis and identified both a substantial mental health burden and promising intervention effects across diverse implementation settings. Overall, the findings reinforce the importance of incorporating psychosocial support into TB treatment programs to address the complex interplay between mental health, social determinants, and treatment outcomes that characterizes the TB–depression syndemic.

### 4.1. Mental Health Burden: An Important but Under-Addressed Dimension

The pooled prevalence of depression among people with TB reported by Duko et al. [9] (45.19%) indicates a substantial psychosocial burden that extends well beyond the biomedical dimensions of the disease. This finding is consistent with the conceptualization of TB and depression as a syndemic, in which poverty, undernutrition, stigma, social isolation, medication side effects, and psychological distress may reinforce one another over time [6,7,9]. Although Duko et al. [9] found a somewhat higher pooled prevalence of depression in MDR-TB than in drug-susceptible TB, that subgroup difference was not statistically significant.

Farooq et al. [25] concluded that psychosocial stressors, particularly stigma, socioeconomic disadvantage, guilt, and fear of contagion, shape the mental health and treatment experiences of people with TB. Taken together, the evidence suggests that TB-related psychological distress is rooted in social meanings and structural vulnerability, reinforcing the need for psychosocial support models that respond to these pressures.

The gender-related difference identified by Duko et al. [9], with a higher pooled prevalence of depression among women than among men, deserves careful consideration in the design of interventions. This pattern may reflect intersecting vulnerabilities, including gendered forms of stigma, caregiving burdens, economic dependence, and barriers to healthcare access. These findings support the development of gender-sensitive psychosocial strategies that acknowledge the different pressures women may face in high-burden settings.

The high heterogeneity of the pooled mental health estimates (I^2^ values ranging from 87% to 99%) points to important methodological variation across studies, including differences in assessment instruments, cultural understandings of mental health, sampling approaches, and population characteristics. Depression was measured with instruments such as the PHQ-9, BDI, HADS, and HDRS, each with different psychometric properties and cut-off scores. This heterogeneity highlights the need for more standardized and culturally validated mental health assessment protocols in TB research and surveillance. 

### 4.2. Integrated Care Models: Evidence of Intervention Effectiveness

Across intervention studies, psychosocial approaches—including cognitive behavioral therapy, acceptance and commitment therapy, motivational enhancement therapy, psychoeducation, and multicomponent psychosocial support—were generally associated with improved psychological outcomes and better adherence-related indicators. For readers who may be unfamiliar with these terms, brief definitions are provided. Cognitive behavioral therapy (CBT) is a structured, time-limited, and evidence-based psychotherapy that helps patients identify and modify dysfunctional thoughts, emotions, and behaviors related to their illness, with the goal of reducing psychological distress and supporting health-related behavior change [38]. Acceptance and commitment therapy (ACT) is a third-wave cognitive-behavioral approach that promotes psychological flexibility through six core processes—acceptance, cognitive defusion, present-moment awareness, self-as-context, values clarification, and committed action—encouraging patients to pursue meaningful goals (such as completing treatment) even in the presence of distressing thoughts or symptoms [39,40]. Motivational enhancement therapy (MET) is a brief, client-centered intervention adapted from motivational interviewing that aims to resolve ambivalence about adopting or sustaining a health behavior, such as adhering to TB medication, by exploring patients’ own motivations, values, and goals [41]. Psychoeducation refers to structured educational interventions that provide information about the disease, treatment, and self-management strategies, often combined with emotional support and skill-building [42]. Multicomponent psychosocial support combines two or more of the above elements with practical assistance such as food parcels, transport vouchers, peer support, or family involvement [16]. Although effect sizes and outcome definitions varied across studies, the overall pattern suggests that addressing mental health and psychosocial barriers may improve treatment engagement in TB care [28,29,30,31,32].

The intervention literature also suggests that adherence can improve when psychosocial support is embedded in broader patient-centered care. For example, Janmeja et al. [28] reported higher treatment success in the intervention group than in the control group, Tola et al. [29] found reduced non-adherence in the intervention arm, and Acha et al. [34] described perceived adherence benefits associated with long-term peer-support participation. These findings are consistent with the End TB Strategy’s emphasis on integrated, patient-centered models of care that address the biological, psychological, social, and economic dimensions of treatment [13].

The multidimensional nature of adherence barriers is also reflected in the apparent value of multicomponent interventions combining psychological support, education, and material or social assistance [16,19,36]. In Yin et al. [33], financial and social support were associated with improved treatment outcomes, and perceived social support appeared to mediate part of the relationship between financial assistance and treatment success. Taken together, these findings suggest that social protection and psychosocial support are best understood as complementary components of person-centered TB care rather than as optional add-ons.

### 4.3. Implementation Science Insights: Feasibility and Scalability

One important practical implication is that psychosocial interventions do not always need to be delivered by specialist mental health professionals. In the trial by Zuo et al. [30], trained general practitioners delivered cognitive behavioral therapy in community settings, suggesting that task-shifting approaches may be feasible in resource-constrained contexts. This aligns with the World Health Organization’s mhGAP framework, which supports expanding mental health care through trained non-specialist providers where specialist capacity is limited [17].

At the same time, qualitative findings from Yin et al. [33] highlight the gap that can emerge between intervention design and real-world implementation fidelity. In that study, family-member treatment observers were often insufficiently trained and monitored only a limited proportion of doses. Kaliakbarova et al. [36] likewise described operational challenges, including heavy workloads, resource constraints, and implementation demands that could undermine intervention delivery despite good intentions and financial support.

The sustainability issues described by Kaliakbarova et al. [36]—including increased budgetary allocations, the institutionalization of psychologist and social worker roles, and the creation of a government-led working group—suggest that successful integration of psychosocial interventions depends not only on pilot effectiveness, but also on political commitment, dedicated funding, workforce development, and institutional capacity. More broadly, studies such as Munteanu et al. [19] illustrate the trade-offs typical of pragmatic implementation research: real-world relevance may increase, while causal certainty remains limited when control groups are absent.

### 4.4. High-Risk Groups: The Need for Tailored Interventions

The markedly poorer treatment outcomes reported among socially vulnerable groups, such as people who inject drugs and people experiencing homelessness [19], suggest that generalized support models may be insufficient for populations facing layered forms of disadvantage. These groups often contend with substance-use problems, mental illness, unstable housing, social disconnection, and structural exclusion. Likewise, Dhumal et al. [18] showed that adolescents and young adults with MDR-TB expressed preferences for digital resources, peer counseling, and flexible delivery formats. Together, these findings support the need for tailored rather than one-size-fits-all psychosocial strategies. Several specific strategies have shown promise for engaging these populations in psychosocial interventions. For people who inject drugs and people with harmful alcohol use, integrating TB care with harm-reduction services, opioid agonist therapy, and trained counselors who address substance use and TB simultaneously has been associated with improved treatment retention and adherence [43]. For people experiencing homelessness, community- and outreach-based delivery—such as nurse–community-health-worker teams that meet patients in shelters or on the street—has been shown to improve engagement with TB and latent TB infection treatment while also reducing drug use [44,45]. Building rapport and trust through low-threshold, non-stigmatizing services in community-based organizations, rather than in large institutional clinics, helps mitigate the anticipated discrimination that often deters these patients from seeking care [46]. Additional engagement strategies supported by the reviewed literature include the use of incentives and enablers (e.g., transport vouchers, food parcels, hygiene kits), peer support workers with lived experience of TB or substance use, multidisciplinary teams that combine clinical, psychological, and social work roles, and culturally tailored education delivered at multiple levels (patient, family, provider, community) [15,36,47]. For adolescents and young adults, digital and mobile health components, brief peer-led psychoeducation, and flexible scheduling are particularly aligned with patient preferences and feasible in resource-constrained settings [18]. 

### 4.5. Stigma as a Cross-Cutting Barrier

Stigma emerged across the literature as both a mental health determinant and a barrier to adherence. It may contribute to depression and anxiety through internalized shame and experiences of discrimination; delay help-seeking because of fear of disclosure; interfere with adherence by making treatment-taking visible; and intensify social isolation through both self-protective withdrawal and rejection by others [6,27,35]. This helps explain why stigma-reduction strategies increasingly target multiple levels, including self-stigma, anticipated stigma, healthcare-provider attitudes, community norms, and structural discrimination.

The findings that even trained staff continued to use stigmatizing language and that mental health was sometimes interpreted as a sign of personal weakness rather than a treatable condition [35] highlight the depth of attitudinal barriers in TB care. Sustained, culturally grounded mental health literacy efforts are needed for patients, healthcare workers, policymakers, and communities.

### 4.6. Theoretical Models to Guide Intervention Design

The apparent usefulness of established behavioral frameworks—including the Health Belief Model [29,32], Motivational Enhancement Therapy drawing on stages-of-change principles [28], and the Capability, Opportunity, Motivation, and Behavior (COM-B) model and Behavior Change Technique Taxonomy [20]—suggests that theory-informed intervention design may strengthen psychosocial programs by linking strategies to modifiable determinants of behavior. In the specific case of TB care, the Health Belief Model proposes that adherence is shaped by patients’ perceived susceptibility to TB-related complications, perceived severity of the disease, perceived benefits of completing treatment, perceived barriers to taking medication regularly (such as side effects, stigma, or transport costs), cues to action, and self-efficacy in managing the regimen [29,48]. When used to guide intervention design, these constructs are operationalized into concrete counseling components: educational sessions that strengthen perceived susceptibility and severity by clarifying transmission and complication risks; messages that highlight the personal and family-level benefits of cure; structured problem-solving and adherence aids that reduce perceived barriers; reminders and family or peer encouragement that act as cues to action; and skills training and goal setting that build self-efficacy [32,49]. The cluster randomized trial by Tola et al. [29] illustrates this approach: a 7-session counseling and education protocol mapped onto Health Belief Model domains reduced TB treatment non-adherence from 19.4% to 9.5% in the intervention group (AOR = 0.31, *p* < 0.001), supporting the value of explicit theoretical mapping in psychosocial programs. At the same time, critiques of individually focused models, such as the Health Belief Model, indicate that broader frameworks incorporating social context, structural determinants, and syndemic interactions may be better suited to the realities of TB care.

### 4.7. Health Policy and Clinical Implications

These findings support strengthening TB control efforts by complementing biomedical treatment with person-centered approaches that address the psychological, social, and economic dimensions of illness. Routine mental health screening using validated tools, together with clear referral pathways for those needing more intensive care, should be considered within TB services. Short-term psychosocial interventions—such as cognitive behavioral therapy, acceptance and commitment therapy, and psychoeducation—may also be integrated into existing TB programs through trained non-specialist providers, thereby improving accessibility while making efficient use of limited resources.

### 4.8. Limitations

This review has several limitations. As a scoping review, its purpose was to map and synthesize the available evidence rather than to calculate pooled intervention effects. Because the included studies varied widely in design, population, intervention type, and outcome measures, a meta-analysis of intervention effectiveness was not appropriate. The findings should therefore be interpreted as a narrative synthesis of a heterogeneous evidence base.

The search was limited to PubMed and Scopus and English-language publications or those with translations available between 2005 and 2025. Relevant studies indexed elsewhere or published in other languages may therefore have been missed. In addition, gray literature and unpublished program evaluations were not systematically included, which may mean that real-world implementation efforts—especially in low-resource settings—are underrepresented. Some primary studies, particularly program evaluations and observational designs, lacked randomized control groups, limiting the strength of causal conclusions. Long-term follow-up data were also scarce, making it difficult to determine whether psychosocial benefits and adherence improvements are sustained beyond treatment completion.

Despite these limitations, this review identifies consistent patterns across diverse settings that support the integration of psychosocial care into tuberculosis services. Future research should prioritize standardized outcome measures, longer follow-up periods, and rigorous evaluation of scalable, context-sensitive intervention models.

## 5. Conclusions

This scoping review suggests that psychosocial interventions are important components of person-centered TB care and may help improve treatment adherence and related outcomes. Across the 19 included studies, depression and anxiety emerged as common mental health problems among people with TB, with additional burden documented in MDR-TB populations. Interventions such as cognitive behavioral therapy, acceptance and commitment therapy, motivational enhancement therapy, psychoeducation, and multicomponent psychosocial support were associated with improved psychological outcomes and better adherence-related indicators in several settings. Taken together, these findings support a shift from disease-centered models toward integrated TB care that includes routine mental health attention, scalable evidence-based psychosocial support, and implementation strategies such as task-shifting where appropriate. Future priorities include culturally appropriate assessment, stronger stigma-reduction and social-protection components, and more rigorous implementation and cost effectiveness research to guide decision-making.

## Figures and Tables

**Table 1:** Database-specific Boolean search strategies.

Database	Search Strategy (String)	Filters/Limits
PubMed	(tuberculosis [MeSH Terms] OR tuberculosis [Title/Abstract] OR TB [Title/Abstract] OR “pulmonary tuberculosis” [Title/Abstract] OR MDR-TB [Title/Abstract] OR “multidrug-resistant tuberculosis” [Title/Abstract] OR “drug-resistant tuberculosis” [Title/Abstract] OR RR-TB [Title/Abstract]) AND (psychosocial [Title/Abstract] OR “mental health” [Title/Abstract] OR depression [Title/Abstract] OR depressive [Title/Abstract] OR anxiety [Title/Abstract] OR anxious [Title/Abstract] OR “psychological distress” [Title/Abstract] OR stress [Title/Abstract] OR stigma [Title/Abstract] OR “social support” [Title/Abstract] OR “quality of life” [Title/Abstract] OR counseling [Title/Abstract] OR psychoeducation [Title/Abstract] OR psychotherapy [Title/Abstract] OR “motivational enhancement” [Title/Abstract] OR CBT [Title/Abstract] OR “cognitive behavioral therapy” [Title/Abstract] OR ACT [Title/Abstract] OR “acceptance and commitment therapy” [Title/Abstract]) AND (adherence [Title/Abstract] OR compliance [Title/Abstract] OR default* [Title/Abstract] OR “treatment interruption” [Title/Abstract] OR “treatment completion” [Title/Abstract] OR “treatment success” [Title/Abstract] OR outcome* [Title/Abstract])	Humans; English; Publication date from 1 January 2005 to 31 December 2025
Scopus	TITLE-ABS-KEY (tuberculosis OR TB OR “pulmonary tuberculosis” OR MDR-TB OR “multidrug-resistant tuberculosis” OR “drug-resistant tuberculosis” OR RR-TB) AND TITLE-ABS-KEY (psychosocial OR “mental health” OR depress* OR anxi* OR “psychological distress” OR stress* OR stigma* OR “social support” OR “quality of life” OR counseling OR psychoeducation OR psychotherapy OR “motivational enhancement” OR CBT OR “cognitive behavioral therapy” OR ACT OR “acceptance and commitment therapy”) AND TITLE-ABS-KEY (adherence OR compliance OR default* OR “treatment interruption” OR “treatment completion” OR “treatment success” OR outcome*)	PUBYEAR > 2004 AND PUBYEAR < 2026 (equivalent to 2005–2025)

**Note:** The asterisk (*) indicates truncation and was used to capture word variations, such as depress* for depression, depressive, and depressed; and anxi* for anxiety and anxious.

**Table 2:** Pooled prevalence of mental health outcomes among TB and MDR-TB populations.

Author	Publication Year	Mental Health Outcome	Study Population	Pooled Prevalence (95% CI)	Heterogeneity (I^2^)
Alene et al. [7]	2018	Depression	MDR-TB patients (15 studies)	25% (14–39)	98%
Alene et al. [7]	2018	Anxiety	MDR-TB patients (3 studies)	24% (2–57)	96%
Alene et al. [7]	2018	Psychosis	MDR-TB patients (12 studies)	10% (7–14)	87%
Duko et al. [9]	2020	Depression (overall)	All TB patients (25 studies; *n* = 4903)	45.19% (38.04–52.55)	96.28%
Duko et al. [9]	2020	Depression (DS-TB)	Drug-susceptible TB (20 studies)	43.47% (35.88–51.37)	99.26%
Duko et al. [9]	2020	Depression (MDR-TB)	Multidrug-resistant TB (5 studies)	52.34% (38.09–66.22)	92.55%

CI = confidence interval; DS-TB = drug-susceptible TB; MDR-TB = multidrug-resistant TB.

**Table 3:** Characteristics of Included Studies.

No.	Author/Paper/Country	Type of Study	Sample	Research Objectives	Main Instrument and Measures	Intervention	Main Result
1	Sweetland et al. [6] Global/Multinational including Latin America, Sub-Saharan Africa, and South Asia	Review/Perspective piece reframing comorbidity as a syndemic	Narrative synthesis (including Peru cohort of 285 MDR-TB patients as a case study)	Reframe TB and depression as a syndemic to inform integrated care	Synthesis of biological, social, and behavioral mechanisms	Integrated care models, collaborative care, and psychosocial support	Conceptualized TB and depression as interacting syndemics, highlighting reciprocal effects of poverty, stigma, and mental illness on TB outcomes and arguing for integrated care models.
2	Alene et al. [7] 20 countries	Systematic review and meta-analysis	40 quantitative studies involving MDR-TB patients	Quantify mental health disorders, social stressors, and HRQoL in MDR-TB patients	Systematic search and random-effects meta-analysis	Recommends integrating mental health services and social protection into clinical management	Meta-analysis demonstrated high pooled prevalence of mental health disorders and social stressors among MDR-TB patients, supporting the need for integration of mental health services within MDR-TB care.
3	Duko et al. [9] 7 countries	Systematic review and meta-analysis	25 studies including 4903 participants with TB	Quantitatively summarize prevalence of depression among patients with TB	Meta-analysis of outcomes from PHQ-9, HADS, BDI, and HDRS scales	Recommends integration of depression screening and management within TB services.	Meta-analysis estimated a pooled prevalence of depression of approximately 45% among TB patients, with higher prevalence in MDR-TB populations.
4	Dhumal et al. [18] India	Mixed-method study	81 (6 qualitative + 75 quantitative), but main reported dataset = 75 adolescents and young adults (AYA) with MDR-TB	Document psychosocial challenges and identify acceptable support interventions	In-depth interviews, semi-structured questionnaire, and DSM-5 scale	Preferred interventions: social media awareness, deep breathing, and exercise training	Adolescents and young adults with MDR-TB reported high psychological distress and stigma; participants expressed preference for accessible counseling and peer-support interventions.
5	Munteanu et al. [19] Romania	Retrospective cohort study	4104 patients from disadvantaged groups (rural, drug users, homeless)	Identify predictors of therapeutic success through multidisciplinary support	Multivariate logistic regression; ISCED education levels	Multidisciplinary teams providing cash subsidies, peer support, and social assistance	Peer-to-peer support and social assistance were strong predictors of therapeutic success among disadvantaged TB patients (Exp(B) = 3.742, *p* < 0.001).
6	Viegas et al. [21] Portugal	Qualitative study	17 adult TB patients	Explore multifaceted patient experiences and Patient-Reported Outcomes (PROs)	Semi-structured interviews; inductive and deductive thematic analysis	Recommends nutritional support and multidisciplinary care to improve outcomes	TB patients reported treatment fatigue, medication side effects, economic hardship, and social isolation; authors recommended nutritional and multidisciplinary psychosocial support to improve patient-reported outcomes.
7	Farooq et al. [25] Multiple	Systematic review	13 studies: TB, MDR-TB, staff, and nurses	Review literature on interventions for treating Common Mental Disorders (CMD) in TB	PRISMA guidelines; Cochrane and Newcastle-Ottawa risk of bias tools	Psychosocial (CBT, ACT, counseling) and pharmacological (Imipramine, Vitamin D) interventions	High prevalence of depression and anxiety was observed among TB patients, significantly associated with lower treatment adherence.
8	Cannon et al. [26] Multiple African Countries	Scoping review	22 articles (17 quantitative, 3 reviews, 2 additional studies)	Identify socio-economic drivers contributing to the burden of DR-TB in Africa	JBI scoping review approach using the PCC framework	Focus on socio-economic empowerment and holistic interventions	Socioeconomic factors including poverty, stigma, overcrowding, and substance use were consistently associated with poor MDR-TB treatment outcomes across African studies.
9	Aitambayeva et al. [27] Multiple	Systematic review	15 studies; descriptive review	Identify and synthesize evidence for TB stigma reduction interventions	Mixed Methods Appraisal Tool (MMAT) and PRISMA guidelines	Educational programs, video-based therapy, peer-led support, and self-stigma toolkits	TB patients reported stigma, fear of disclosure, and social rejection, contributing to emotional distress and delayed care-seeking.
10	Janmeja et al. [28] India	Prospective single-blind, controlled trial	200 TB outpatients (100 intervention, 100 control)	Evaluate behavior modification via psychotherapy to improve treatment compliance	Hamilton Rating Scale (Anxiety/Depression); PGI Health Questionnaire	8 sessions of individual psychotherapy based on Motivational Enhancement Therapy	Motivational Enhancement Therapy-based psychotherapy improved TB treatment outcomes, with higher treatment success and lower default in the intervention group compared with standard care.
11	Tola et al. [29] Ethiopia	Cluster randomized controlled trial	698 TB patients from 30 randomly selected Health Centers	Evaluate impact of psychological counseling and education on adherence using the Health Belief Model (HBM)	Structured questionnaire (HBM domains), Kessler-10 (K-10), AUDIT, and Visual Analogue Scale (VAS)	7 sessions of counseling and adherence education targeting HBM domains	Health Belief Model-based counseling and education reduced treatment non-adherence compared with routine care, supporting integration into TB services.
12	Zuo et al. [30] China	Community-based cluster randomized controlled trial	454 pulmonary TB patients (230 intervention, 224 control)	Explore the effects of Cognitive Behavioral Therapy (CBT) on psychological stress and QoL	PHQ-9, GAD-7, and SF-36 scales	8 weekly CBT lessons delivered by general practitioners	Community-delivered CBT (8 weekly sessions over 2 months) reduced anxiety and depression symptoms compared with usual care; between-group differences at 2 months were significant (e.g., GAD-7 lower by ~1.72 points and PHQ-9 lower by ~2.05 points in the CBT group; *p* < 0.001).
13	Suryani et al. [31] Indonesia	Quasi-experimental study (pre–post test with control group)	74 TB patients (37 intervention, 37 control)	Test the effect of psychoeducation on levels of depression, anxiety, and stress	Depression Anxiety Stress Scale (DASS) questionnaire	Combined active (counseling) and passive (booklets) psychoeducation	Psychoeducation reduced psychological distress, with substantially more participants achieving normal DASS scores in the intervention group compared with controls.
14	Sari et al. [32] Indonesia	Quantitative quasi-experiment (pre-posttest design)	56 pulmonary TB respondents	Explain the effect of Acceptance and Commitment Therapy (ACT) on depression	Beck Depression Inventory-II (BDI-II) questionnaire	4 sessions of ACT (30–45 min) via direct counseling	ACT based on the Health Belief Model significantly reduced depression scores (BDI-II) in the intervention group (mean 15.75 → 5.96; Δ = −9.79), with a greater reduction than the control group (Δ = −7.35); between-group difference significant (*p* = 0.001).
15	Yin et al. [33] China	Retrospective cohort and mixed-method study	218 patients with MDR-TB	Examine the relationship between social support and MDR-TB treatment outcomes	Social Support Rating Scale (SSRS), pathway analysis, and in-depth interviews	Directly Observed Therapy (DOT), financial support ($10/month), and health education	Financial and social support were associated with improved adherence and treatment success among MDR-TB patients, with perceived social support mediating part of the effect of financial assistance.
16	Acha et al. [34] Peru	Detailed case history and qualitative study	285 MDR-TB patients	Trace the first 5 years of a psychosocial support group aimed at improving treatment adherence	Participant observation, informal focus groups, session summaries, and transcripts	Bi-monthly support groups, recreational excursions, symbolic celebrations, and family workshops	Five years of facilitated TB patient support groups revealed persistent emotional distress, social isolation, stigma, and economic hardship among participants, while peer-group participation provided perceived emotional support and coping benefits.
17	Mainga et al. [35] Zambia	Qualitative study	148 participants (57 IDIs; 91 FGDs)	Understand distress in TB	Focus Group Discussions (FGDs) and in-depth interviews (IDIs)	N/A (investigative qualitative study)	Health workers and research staff continued to use stigmatizing language for mental illness, and mental health was commonly conceptualized as character weakness rather than a treatable condition, indicating entrenched stigma in TB care settings.
18	Kaliakbarova et al. [36] Kazakhstan	Analysis of a pilot psychosocial support (PSS) program	228 MDR-TB patients at high risk of treatment default	Assess the effects of Psychosocial Support on default rates and treatment adherence	Two rounds of interviews, medical records, and psychological profiles	Support packages (food/hygiene), psychological counseling, and assistance with documentation or housing	Implementation of a national psychosocial support program for MDR-TB patients was associated with improved treatment adherence and reduced default during program rollout.
19	Araújo et al. [37] Brazil	Matched case–control study	1434 individuals (717 TB cases and 717 controls)	Investigate the association between common mental disorders (CMDs) and pulmonary TB	Self-Reporting Questionnaire (SRQ-20) and CAGE for alcoholism	Observational study (no specific intervention evaluated)	Common mental disorders were significantly associated with active TB, with higher odds of TB among participants screening positive for mental disorder (OR 1.34; 95% CI 1.05–1.70).

ACT = Acceptance and Commitment Therapy; AUDIT = Alcohol Use Disorder Identification Test; AYA = Adolescents and Young Adults; BDI/BDI-II = Beck Depression Inventory/Beck Depression Inventory-II; CAGE = Cut Down, Annoyed, Guilty, and Eye-Opener; CBT = Cognitive Behavioral Therapy; CMD/CMDs = Common Mental Disorder(s); DASS = Depression, Anxiety, and Stress Scale; DOT = Directly Observed Therapy; DR-TB = Drug-Resistant Tuberculosis; DSM-5/DSM-IV = Diagnostic and Statistical Manual of Mental Disorders, Fifth/Fourth Edition; FGDs = Focus Group Discussions; GAD-7 = Generalized Anxiety Disorder-7; HADS = Hospital Anxiety and Depression Scale; HBM = Health Belief Model; HDRS = Hamilton Depression Rating Scale; HRQoL = Health-Related Quality of Life; IDIs = In-Depth Interviews; ISCED = International Standard Classification of Education; JBI = Joanna Briggs Institute; K-10 = Kessler-10 Psychological Distress Scale; MDR-TB = Multidrug-Resistant Tuberculosis; MMAT = Mixed Methods Appraisal Tool; PCC = Population, Concept, and Context; PGI Health Questionnaire = Postgraduate Institute Health Questionnaire; PHQ-9 = Patient Health Questionnaire-9; PRISMA = Preferred Reporting Items for Systematic Reviews and Meta-Analyses; PROs = Patient-Reported Outcomes; PSS/PSSG = Psychosocial Support (program/group); SF-36/SF-8 = 36-Item/8-Item Short-Form Health Survey; SRQ-20 = Self-Reporting Questionnaire-20; SSRS = Social Support Rating Scale; TB = Tuberculosis; VAS = Visual Analogue Scale.

**Table 4:** Primary Psychosocial Intervention Studies for TB Treatment Adherence.

Study	Country	Design	Sample (*n*)	Intervention Type	Duration	Primary Outcome	Effect Size/ Results	Quality
Janmeja et al. [28]	India	Prospective single-blind, controlled trial	200 outpatients (100 intervention, 100 control)	Individual psychotherapy based on Motivational Enhancement Therapy (MET)	6 months (8 sessions: intensive for 2 months, monthly for 4 months)	Treatment compliance and successful treatment rate	Successful treatment was 83% in the intervention group vs. 47% in the control (*p* < 0.001)	Moderate (Controlled trial)
Tola et al. [29]	Ethiopia	Cluster randomized control trial (RCT)	698 TB patients (368 intervention, 330 control)	HBM-based therapy: Counseling and education based on the Health Belief Model	4 months (7 sessions)	Treatment non-adherence level (measured via VAS)	Non-adherence decreased from 19.4% to 9.5% in the intervention group (AOR = 0.31, *p* < 0.001)	High (Cluster RCT)
Zuo et al. [30]	China	Community-based cluster randomized controlled trial	454 pulmonary TB patients	CBT: Cognitive Behavioral Therapy delivered by general practitioners	2 months (8 weekly lessons)	Anxiety (GAD-7), Depression (PHQ-9), and Quality of Life (SF-36)	Significant relief in anxiety/depression; CBT group scores were ~2 points lower than control (*p* < 0.001)	High (Cluster RCT)
Suryani et al. [31]	Indonesia	Quasi-experimental (pre-post with control group)	74 pulmonary TB patients (37 per group)	Psychoeducation: Combined active counseling and passive booklets	5-month study period; 1-week post-intervention follow-up	Levels of depression, anxiety, and stress (DASS)	Significant reduction in symptoms; 75.7% reached “normal” levels post-intervention	Moderate (Quasi-experimental)
Sari et al. [32]	Indonesia	Quantitative quasi-experiment (pre-posttest control)	56 pulmonary TB patients (28 per group)	ACT: Acceptance and Commitment Therapy based on HBM.	4 sessions (once per week), 30–45 min each	Depression level (measured via BDI-II)	Significant drop in depression; intervention delta (−9.79) was higher than control (*p* = 0.001)	Moderate (MMAT)
Kaliakbarova et al. [36]	Kazakhstan	Programmatic pilot evaluation (non-randomized)	228 MDR-TB patients at high risk of default	Holistic PSS: Psychological counseling, food/hygiene parcels, and social assistance	Throughout treatment duration	Patient default rates and adherence	Program implementation was associated with reduced default rates and improved dose adherence during program rollout.	Moderate (Pilot evaluation)

ACT = Acceptance and Commitment Therapy; BDI-II = Beck Depression Inventory-II; CBT = Cognitive Behavioral Therapy; DASS = Depression, Anxiety, and Stress Scale; GAD-7 = Generalized Anxiety Disorder-7; HBM = Health Belief Model; MDR-TB = multidrug-resistant tuberculosis; PHQ-9 = Patient Health Questionnaire-9; VAS = Visual Analogue Scale.

**Table 5:** Psychosocial Intervention Delivery Characteristics.

Delivery Feature	*n*	%	Examples
Setting			
Community-based	4	50%	Interventions in metropolitan Lima (Acha et al. [34]); Pilot support in East Kazakhstan cities (Kaliakbarova et al. [36]); Outreach in Romanian rural areas (Munteanu et al. [19]); Trial across 20 Chinese communities (Zuo et al. [30])
Hospital/clinic-based	4	50%	Outpatient departments in Chandigarh (Janmeja et al. [28]); primary health care centers in Sidoarjo (Sari et al. [32]); Garuda and Babakan Sari health centers (Suryani et al. [31]); 30 randomly selected health centers in Addis Ababa (Tola et al. [29]).
Provider Type			
Multidisciplinary team	3	37.5%	Teams of psychiatrists and nurses (Acha et al. [34]); psychologists, social workers, and TB nurses (Kaliakbarova et al. [36]); multidisciplinary teams providing psychological and social assistance (Munteanu et al. [19])
Psychologist/psychiatrist	1	12.5%	Therapy performed by a qualified clinical psychologist (Janmeja et al. [28]).
Trained general practitioners	1	12.5%	CBT delivered by community GPs after an intensive 8 h skills training session (Zuo et al. [30])
Nurses/community health workers	3	37.5%	Interventions led by registered nurses (Sari et al. [32]); Health education by health center nurses (Suryani et al. [31]); Trained health professionals at TB clinics (Tola et al. [29])
Session Frequency			
Weekly (intensive phase)	3	37.5%	4 sessions once a week (Sari et al. [32]); Weekly for the first month of enrolment (Tola et al. [29]); 8 weekly lessons over two months (Zuo et al. [30])
Bi-monthly	1	12.5%	Facilitated MDR-TB patient support groups held twice per month over an extended treatment period (Acha et al. [34])
Monthly (continuation phase)	2	25%	Sessions held at monthly intervals during the continuation phase (Janmeja et al. [28]); Once a month until the end of follow-up (Tola et al. [29]).
Not clearly session-based/ongoing	2	25%	Long-term PSS integrated throughout MDR-TB treatment (Kaliakbarova et al. [36]); Multidisciplinary psychosocial support provided across the full treatment course without fixed session frequency (Munteanu et al. [19])
Intervention Duration			
<1 month	1	12.5%	Brief intervention consisting of 4 sessions over 4 weeks (Sari et al. [32])
2–6 months	4	50%	Combined psychotherapy over a 6-month treatment course (Janmeja et al. [28]); Short-term study period (Suryani et al. [31]); Counseling and education for 4 months (Tola et al. [29]); CBT lessons delivered over 2 months (Zuo et al. [30])
>6 months	3	37.5%	Support provided over 5 years of experience with 2-year MDR-TB regimens (Acha et al. [34]); Long-term PSS program implementation for MDR-TB (Kaliakbarova et al. [36]); Multidisciplinary support spanning the full treatment course (Munteanu et al. [19])

## Data Availability

Not applicable.

## Supplementary Materials

The following supporting information can be downloaded at: https://www.mdpi.com/article/10.3390/arm94030032/s1, Figure S1, high-resolution PRISMA-ScR flow diagram of study identification, screening, and inclusion; Table S1, quality appraisal summary table; Table S2, preferred Reporting Items for Systematic reviews and Meta-Analyses extension for Scoping Reviews (PRISMA-ScR) Checklist; Table S3, planned but not executed database-specific Boolean search strategies.

## Abbreviations

The following abbreviations are used in this manuscript:

ACT	Acceptance and Commitment Therapy
AOR	Adjusted Odds Ratio
AUDIT	Alcohol Use Disorder Identification Test
AYA	Adolescents and Young Adults
BDI/BDI-II	Beck Depression Inventory/Beck Depression Inventory-II
CAGE	Cut Down, Annoyed, Guilty, and Eye-Opener (alcohol-use screening tool)
CBT	Cognitive Behavioral Therapy
CI	Confidence Interval
CMD/CMDs	Common Mental Disorders
COM-B	Capability, Opportunity, Motivation, and Behavior (behavior change model)
DASS	Depression, Anxiety, and Stress Scale
DOT	Directly Observed Therapy
DOTS	Directly Observed Treatment, Short-course
DR-TB	Drug-Resistant Tuberculosis
DS-TB	Drug-Susceptible Tuberculosis
DSM-5/DSM-IV	Diagnostic and Statistical Manual of Mental Disorders (5th or 4th Edition)
FGDs	Focus Group Discussions
GAD-7	Generalized Anxiety Disorder-7 scale
HADS	Hospital Anxiety and Depression Scale
HBM	Health Belief Model
HDRS	Hamilton Depression Rating Scale
HRQoL	Health-Related Quality of Life
IDIs	In-depth Interviews
ISCED	International Standard Classification of Education
JBI	Joanna Briggs Institute
K-10	Kessler-10 psychological distress scale
MDR-TB	Multidrug-Resistant Tuberculosis
MET	Motivational Enhancement Therapy
mhGAP	Mental Health Gap Action Programme
MMAT	Mixed Methods Appraisal Tool
OR	Odds Ratio
PCC	Population, Concept, and Context framework
PGI Health Questionnaire	Postgraduate Institute Health Questionnaire (for psychosomatic status)
PHQ-9	Patient Health Questionnaire-9
PRISMA	Preferred Reporting Items for Systematic Reviews and Meta-Analyses
PROs/PROMs	Patient-Reported Outcomes/Patient-Reported Outcome Measures
PSS/PSSG	Psychosocial Support (Program/Group)
RCT	Randomized Controlled Trial
SF-36/SF-8	36-Item/8-Item Short-Form Health Survey
SRQ-20	Self-Reporting Questionnaire-20
SSRS	Social Support Rating Scale
TB	Tuberculosis
TPT	Tuberculosis Preventive Treatment
VAS	Visual Analogue Scale
WHO	World Health Organization

## References

[1] World Health Organization (2024). Global Tuberculosis Report 2024.

[2] Munro S.A., Lewin S.A., Smith H.J., Engel M.E., Fretheim A., Volmink J. (2007). Patient Adherence to Tuberculosis Treatment: A Systematic Review of Qualitative Research. PLoS Med..

[3] Tola H.H., Tol A., Shojaeizadeh D., Garmaroudi G. (2015). Tuberculosis treatment non-adherence and lost to follow up among TB patients with or without HIV in Developing Countries: A systematic review. Iran. J. Public Health.

[4] Alipanah N., Jarlsberg L., Miller C., Linh N.N., Falzon D., Jaramillo E., Nahid P. (2018). Adherence interventions and outcomes of tuberculosis treatment: A systematic review and meta-analysis of trials and observational studies. PLoS Med..

[5] Pablos-Méndez A., Knirsch C.A., Barr R.G., Lerner B.H., Frieden T.R. (1997). Nonadherence in Tuberculosis treatment: Predictors and Consequences in New York City. Am. J. Med..

[6] Sweetland A.C., Kritski A., Oquendo M.A., Sublette M.E., Norcini Pala A., Silva L.R.B., Karpati A., Silva E.C., Moraes M.O., Silva J.R.L.E. (2017). Addressing the tuberculosis–depression syndemic to end the tuberculosis epidemic. Int. J. Tuberc. Lung Dis..

[7] Alene K.A., Clements A.C.A., McBryde E.S., Jaramillo E., Lönnroth K., Shaweno D., Gulliver A., Viney K. (2018). Mental health disorders, social stressors, and health-related quality of life in patients with multidrug-resistant tuberculosis: A systematic review and meta-analysis. J. Infect..

[8] Dos Santos A.P.C., Lazzari T.K., Silva D.R. (2017). Health-Related Quality of Life, Depression and Anxiety in Hospitalized Patients with Tuberculosis. Tuberc. Respir. Dis..

[9] Duko B., Bedaso A., Ayano G. (2020). The prevalence of depression among patients with tuberculosis: A systematic review and meta-analysis. Ann. Gen. Psychiatry.

[10] Anye L.C., Bissong M.E.A., Njundah A.L., Siewe Fodjo J.N. (2023). Depression, anxiety and medication adherence among tuberculosis patients attending treatment centres in Fako Division, Cameroon: Cross-sectional study. BJPsych Open.

[11] Husain M.O., Dearman S.P., Chaudhry I.B., Rizvi N., Waheed W. (2008). The relationship between anxiety, depression and illness perception in tuberculosis patients in Pakistan. Clin. Pract. Epidemiol. Ment. Health.

[12] Pachi A., Bratis D., Moussas G., Tselebis A. (2013). Psychiatric Morbidity and Other Factors Affecting Treatment Adherence in Pulmonary Tuberculosis Patients. Tuberc. Res. Treat..

[13] World Health Organization (2015). The End TB Strategy.

[14] Akyirem S., Forbes A., Wad J.L., Due-Christensen M. (2022). Psychosocial interventions for adults with newly diagnosed chronic disease: A systematic review. J. Health Psychol..

[15] Anindhita M., Haniifah M., Putri A.M.N., Karnasih A., Agiananda F., Yani F.F., Haya M.A.N., Pakasi T.A., Widyahening I.S., Fuady A. (2024). Community-based psychosocial support interventions to reduce stigma and improve mental health of people with infectious diseases: A scoping review. Infect. Dis. Poverty.

[16] Maynard C., Tariq S., Sotgiu G., Migliori G.B., van den Boom M., Field N. (2023). Psychosocial support interventions to improve treatment outcomes for people living with tuberculosis: A mixed methods systematic review and meta-analysis. eClinicalMedicine.

[17] World Health Organization (2023). Mental Health Gap Action Programme (mhGAP) Guideline for Mental, Neurological and Substance Use Disorders.

[18] Dhumal G., Nevrekar N., Gupte N., Shrisunder R., Kulkarni P., Sarpotdar S., Pm A., Hosmani A., Lokhande R., Gupta A. (2025). Improving Treatment Adherence in Youths with Multidrug-Resistant Tuberculosis with Psychosocial Intervention. Brain Behav..

[19] Munteanu I., Kalambayi F., Toth A., Dendrino D., Burdusel B., Vlasceanu S.-G., Parliteanu O., Dragomir A., Nemes R.M., Mahler B. (2025). The Role of Psychosocial Interventions in Increasing Adherence to Tuberculosis Treatment in People Belonging to Socially Vulnerable Categories. Appl. Sci..

[20] Parwitha I.A.A., Djunaidy V.D., Alfian S.D., Setyowibowo H., Pradipta I.S. (2025). Psychosocial interventions to improve tuberculosis preventive treatment uptake and psychosocial outcomes: A systematic review. npj Prim. Care Respir. Med..

[21] Viegas P., Ferreira L.L., Vieira M., Barbosa P., Ramos J.P., Duarte R. (2025). Patient-reported outcomes in tuberculosis: A qualitative exploration of psychosocial, economic, and treatment-related challenges. J. Bras. Pneumol..

[22] Tricco A.C., Lillie E., Zarin W., O’Brien K.K., Colquhoun H., Levac D., Moher D., Peters M.D.J., Horsley T., Weeks L. (2018). PRISMA Extension for Scoping Reviews (PRISMA-ScR): Checklist and Explanation. Ann. Intern. Med..

[23] Falagas M.E., Pitsouni E.I., Malietzis G.A., Pappas G. (2008). Comparison of PubMed, Scopus, Web of Science, and Google Scholar: Strengths and weaknesses. FASEB J..

[24] Gusenbauer M., Haddaway N.R. (2020). Which academic search systems are suitable for systematic reviews or meta-analyses? Evaluating retrieval qualities of Google Scholar, PubMed, and 26 other resources. Res. Synth. Methods.

[25] Farooq S., Tunmore J., Comber R. (2021). Pharmacological or non-pharmacological interventions for treatment of common mental disorders associated with Tuberculosis: A systematic review. Chronic Respir. Dis..

[26] Cannon L.-A.L., Oladimeji K.E., Ter Goon D. (2021). Socio-economic drivers of drug-resistant tuberculosis in Africa: A scoping review. BMC Public Health.

[27] Aitambayeva N., Aringazina A., Nazarova L., Faizullina K., Bapayeva M., Narymbayeva N., Svetlanova S. (2025). A systematic review of tuberculosis stigma reduction interventions. Healthcare.

[28] Janmeja A.K., Das S.K., Bhargava R., Chavan B.S. (2005). Psychotherapy Improves Compliance with Tuberculosis Treatment. Respiration.

[29] Tola H.H., Shojaeizadeh D., Tol A., Garmaroudi G., Yekaninejad M.S., Kebede A., Ejeta L.T., Kassa D., Klinkenberg E. (2016). Psychological and Educational Intervention to Improve Tuberculosis Treatment Adherence in Ethiopia Based on Health Belief Model: A Cluster Randomized Control Trial. PLoS ONE.

[30] Zuo X., Dong Z., Zhang P., Zhang P., Zhu X., Qiao C., Yang Y., Lou P. (2022). Cognitive-behavioral therapy on psychological stress and quality of life in subjects with pulmonary tuberculosis: A community-based cluster randomized controlled trial. BMC Public Health.

[31] Suryani S., Widianti E., Hernawati T., Sriati A. (2016). Psikoedukasi Menurunkan Tingkat Depresi, Stres dan Kecemasan Pada Pasien Tuberkulosis Paru. J. Ners.

[32] Sari G.M., Amin M., Hidayati L. (2020). Acceptance and Commitment Therapy on Depression of Pulmonary Tuberculosis Patient: An Intervention Based on The Health Belief Model. Indones. Nurs. J. Educ. Clin. (INJEC).

[33] Yin J., Wang X., Zhou L., Wei X. (2018). The relationship between social support, treatment interruption and treatment outcome in patients with multidrug-resistant tuberculosis in China: A mixed-methods study. Trop. Med. Int. Health.

[34] Acha J., Sweetland A., Guerra D., Chalco K., Castillo H., Palacios E. (2007). Psychosocial support groups for patients with multidrug-resistant tuberculosis: Five years of experience. Glob. Public Health.

[35] Mainga T., Gondwe M., Stewart R.C., Mactaggart I., Shanaube K., Ayles H., Bond V. (2022). Conceptualization, detection, and management of psychological distress and mental health conditions among people with tuberculosis in Zambia: A qualitative study with stakeholders and TB health workers. Int. J. Ment. Health Syst..

[36] Kaliakbarova G., Pak S., Zhaksylykova N., Raimova G., Temerbekova B., van den Hof S. (2013). Psychosocial Support Improves Treatment Adherence Among MDR-TB Patients: Experience from East Kazakhstan. Open Infect. Dis. J..

[37] Araújo G.S., Pereira S.M., Santos D.N., Marinho J.M., Rodrigues L.C., Barreto M.L. (2014). Common Mental Disorders Associated with Tuberculosis: A Matched Case-Control Study. PLoS ONE.

[38] Hofmann S.G., Asnaani A., Vonk I.J.J., Sawyer A.T., Fang A. (2012). The Efficacy of Cognitive Behavioral Therapy: A Review of Meta-analyses. Cogn. Ther. Res..

[39] Dindo L., Van Liew J.R., Arch J.J. (2017). Acceptance and Commitment Therapy: A Transdiagnostic Behavioral Intervention for Mental Health and Medical Conditions. Neurotherapeutics.

[40] Twohig M.P., Levin M.E. (2017). Acceptance and Commitment Therapy as a Treatment for Anxiety and Depression: A Review. Psychiatry Clin. N. Am..

[41] Lundahl B., Moleni T., Burke B.L., Butters R., Tollefson D., Butler C., Rollnick S. (2013). Motivational interviewing in medical care settings: A systematic review and meta-analysis of randomized controlled trials. Patient Educ. Couns..

[42] Burke A., Davoren M.P., Arensman E., Harrington J.M. (2024). Psychoeducational interventions for people living with chronic communicable disease: A systematic review. BMJ Open.

[43] Harrison R.E., Shyleika V., Falkenstein C., Garsevanidze E., Vishnevskaya O., Lonnroth K., Sayakci Ö., Sinha A., Sitali N., Skrahina A. (2022). Patient and health-care provider experience of a person-centred, multidisciplinary, psychosocial support and harm reduction programme for patients with harmful use of alcohol and drug-resistant tuberculosis in Minsk, Belarus. BMC Health Serv. Res..

[44] Nyamathi A., Morisky D., Wall S.A., Yadav K., Shin S., Hall E., Chang A.H., White K., Arce N., Parsa T. (2022). Nurse-led intervention to decrease drug use among LTBI positive homeless adults. Public Health Nurs..

[45] Pérez Guerrero C.S., Oliveira T.A.C., Bernardi W.O.B., Ribeiro S., Stacciarini J.M., Monroe A.A., Fernandes H., Hino P. (2025). Evidence of tuberculosis treatment outcomes among people experiencing homelessness: A scoping review. BMC Health Serv. Res..

[46] Biancarelli D.L., Biello K.B., Childs E., Drainoni M., Salhaney P., Edeza A., Mimiaga M.J., Saitz R., Bazzi A.R. (2019). Strategies used by people who inject drugs to avoid stigma in healthcare settings. Drug Alcohol Depend..

[47] Ritchie L., Horton J. (2023). Community Supports for People with Tuberculosis: CADTH Health Technology Review.

[48] Azizi N., Karimy M., Salahshour V.N. (2018). Determinants of adherence to tuberculosis treatment in Iranian patients: Application of health belief model. J. Infect. Dev. Ctries..

[49] Parwati N.M., Bakta I.M., Januraga P.P., Wirawan I.M.A. (2021). A Health Belief Model-Based Motivational Interviewing for Medication Adherence and Treatment Success in Pulmonary Tuberculosis Patients. Int. J. Environ. Res. Public Health.

