# Supplementary material for: Psychosocial Interventions for Improving Treatment Adherence in Tuberculosis Patients: A Scoping Review of Evidence-Based Approaches
Source: Adv Respir Med. 2026 May 15;94(3):32. doi: 10.3390/arm94030032 (PMC13214482; doi:10.3390/arm94030032)
Supplement: Supplementary file 1 [file arm-94-00032-s001.zip › Table S1.pdf]

Table S1: Quality Appraisal Summary Table

Study Category	Author & Year	Appraisal Tool	Overall Quality	Key Methodological Note
**Reviews**	Alene et al. (2018)	AMSTAR-2	**High**	PRISMA-compliant; used 5 databases
	Duko et al. (2020)	AMSTAR-2	**High**	Used Comprehensive Meta-Analysis software; checked for bias
	Farooq et al. (2021)	AMSTAR-2	**Moderate**	Descriptive synthesis only due to data heterogeneity
	Cannon et al. (2021)	JBI Scoping Review Checklist	**Moderate**	Scoping approach; focused on African context
	Aitambayeva (2025)	AMSTAR-2	**High**	Intersectional focus; MMAT for internal study appraisal
**RCTs**	Janmeja et al. (2005)	Cochrane RoB	**Moderate**	Single-blind; potential for performance bias
	Tola et al. (2016)	Cochrane RoB	**High**	Cluster RCT; adjusted for 30 health centre clusters
	Zuo et al. (2022)	Cochrane RoB	**High**	Community-based cluster RCT; 98.4% follow-up rate
**Qualitative / Cohort / Mixed**	Acha et al. (2007)	MMAT	**Moderate**	5-year case history; lacks control group
	Kaliakbarova (2013)	MMAT	**Moderate**	Pilot evaluation; effective real-world implementation
	Araújo et al. (2014)	MMAT	**High**	Large matched case-control (n=1,434);

				adjusted for confounders
	Suryani et al. (2016)	MMAT	**Moderate**	Quasi-experimental; 1-week follow-up only
	Sweetland (2017)	Not applicable (Narrative review)	**Conceptual / Low (in evidence hierarchy)**	Syndemic framework; theoretical synthesis
	Yin et al. (2018)	MMAT	**High**	Mixed-methods; clear pathway analysis for support
	Sari et al. (2020)	MMAT	**Moderate**	Small consecutive sample (n=56); brief follow-up
	Mainga et al. (2022)	MMAT	**High**	Large qualitative sample; thematic saturation confirmed
	Dhumal et al. (2025)	MMAT	**Moderate**	Novel youth focus; limited by convenience sampling
	Munteanu et al. (2025)	MMAT	**High**	Large-scale cohort; strong multivariate modelling
	Viegas et al. (2025)	MMAT	**High**	Qualitative; achieved meaning saturation in high-income setting

REPORTS:

A. Systematic and Scoping Reviews (AMSTAR-2)

• Strengths: Reviews by Alene (2018) and Duko (2020) demonstrate high methodological rigor, including protocol registration, comprehensive multi-database searches, and quantitative meta-analytic synthesis. Aitambayeva (2025) adds theoretical depth by mapping stigma-reduction interventions to the COM-B behavioural framework.

• Weaknesses: Farooq (2021) and Cannon (2021) were rated Moderate due to descriptive synthesis designs and limited quantitative pooling.

B. Randomised Controlled Trials (Cochrane Risk of Bias)

• Low Risk of Bias: Tola (2016) and Zuo (2022) are exemplary for using cluster-randomisation, which prevents "information contamination" between different health clinics. Zuo (2022) also verified implementation fidelity by recording and evaluating 24% of the CBT sessions.

• Moderate Risk: Janmeja (2005) utilized a single-blind design where the investigators were blinded but patients knew they received psychotherapy, which can introduce performance bias in self-reported adherence.

C. Qualitative and Observational Studies (MMAT)

• High Rigour: Araújo (2014) adjusted for eight potential confounders including diabetes and alcohol use, providing high-quality evidence of the link between CMDs and active TB. Munteanu (2025) and Viegas (2025) are strong because they utilize multivariate regression or meaning saturation to ensure findings represent the true patient experience.

• Limited Generalisability: Dhumal (2025) and Sari (2020) were rated "Moderate" due to convenience sampling or small sample sizes (n=75 and n=56, respectively), which limits how well their findings apply to broader global populations
