# Supplementary material for: Psychosocial Interventions for Improving Treatment Adherence in Tuberculosis Patients: A Scoping Review of Evidence-Based Approaches
Source: Adv Respir Med. 2026 May 15;94(3):32. doi: 10.3390/arm94030032 (PMC13214482; doi:10.3390/arm94030032)

Supplementary Figure S1: High-resolution PRISMA-ScR flow diagram of study identification, screening and inclusion.

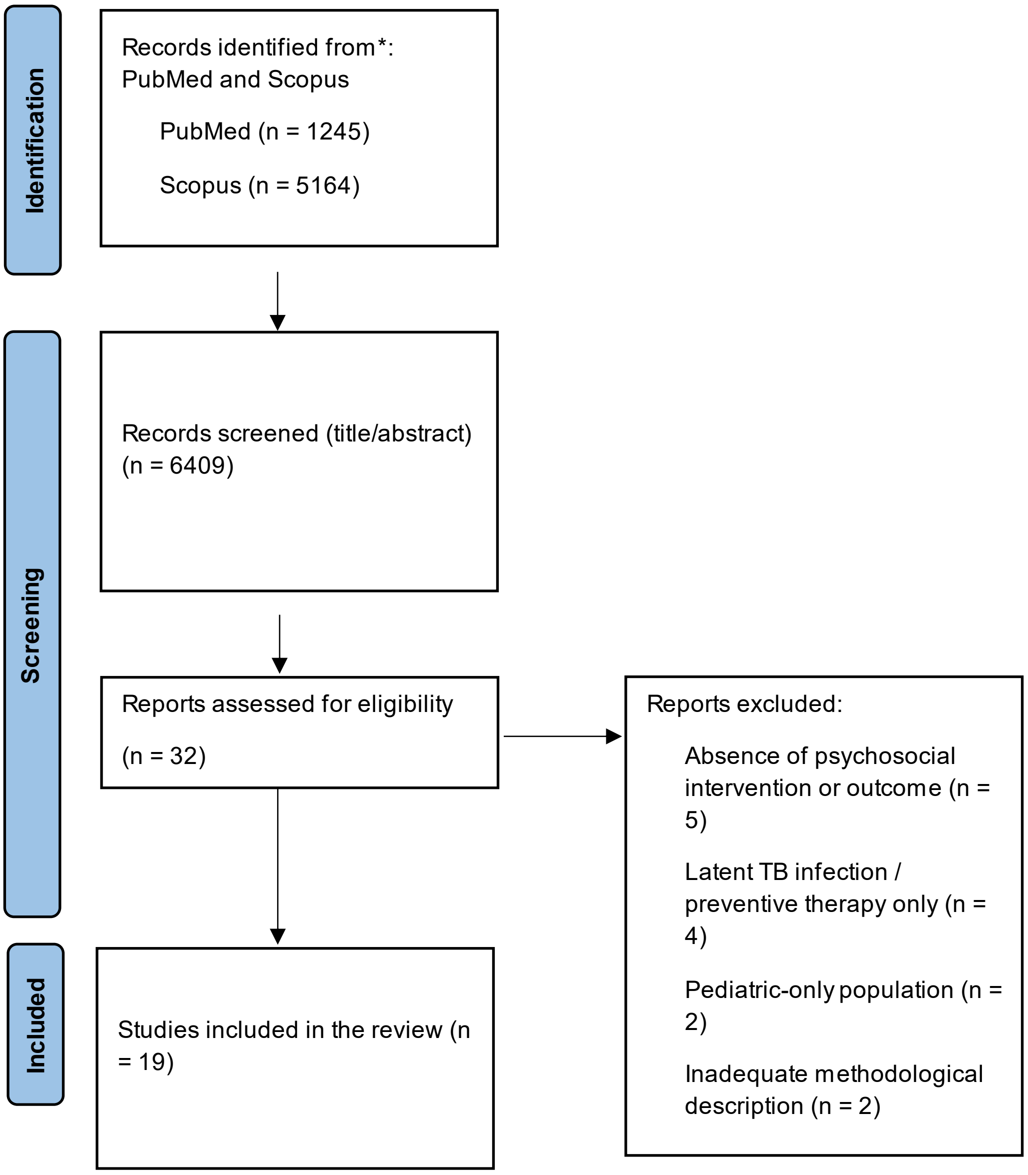

Supplement: Supplementary file 1 [file arm-94-00032-s001.zip › PRISMA Figure S1.pdf]
