# Supplementary material for: Psychosocial Interventions for Improving Treatment Adherence in Tuberculosis Patients: A Scoping Review of Evidence-Based Approaches
Source: Adv Respir Med. 2026 May 15;94(3):32. doi: 10.3390/arm94030032 (PMC13214482; doi:10.3390/arm94030032)
Supplement: Supplementary file 1 [file arm-94-00032-s001.zip › Table S3.pdf]

Table S3: Planned but Not Executed Database-Specific Boolean Search Strategies

Web of Science	TS=(tuberculosis OR TB OR "pulmonary tuberculosis" OR MDR-TB OR "multidrug-resistant tuberculosis" OR "drug-resistant tuberculosis" OR RR-TB) AND TS=(psychosocial OR "mental health" OR depress* OR anxi* OR "psychological distress" OR stress* OR stigma* OR "social support" OR "quality of life" OR counseling OR psychoeducation OR psychotherapy OR "motivational enhancement" OR CBT OR "cognitive behavioral therapy" OR ACT OR "acceptance and commitment therapy") AND TS=(adherence OR compliance OR default* OR "treatment interruption" OR "treatment completion" OR "treatment success" OR outcome*)	Timespan: 2005-2025; English
PsycINFO (EBSCOhost)	(TI(tuberculosis OR TB OR MDR-TB OR "drug-resistant tuberculosis") OR AB(tuberculosis OR TB OR MDR-TB OR "drug-resistant tuberculosis")) AND (TI(psychosocial OR "mental health" OR depress* OR anxi* OR "psychological distress" OR stigma* OR "social support" OR counseling OR psychoeducation OR psychotherapy OR CBT OR "cognitive behavioral therapy" OR ACT OR "acceptance and commitment therapy") OR AB(psychosocial OR "mental health" OR depress* OR anxi* OR "psychological distress" OR stigma* OR "social support" OR counseling OR psychoeducation OR psychotherapy OR CBT OR "cognitive behavioral therapy" OR ACT OR "acceptance and commitment therapy")) AND (TI(adherence OR compliance OR default* OR "treatment interruption" OR "treatment completion" OR "treatment success") OR AB(adherence OR compliance OR default* OR "treatment interruption" OR "treatment completion" OR "treatment success"))	Humans; English; 2005-2025
